# A Generative View of Rationality and Growing Awareness^†^

**DOI:** 10.3389/fpsyg.2022.807261

**Published:** 2022-04-07

**Authors:** Teppo Felin, Jan Koenderink

**Affiliations:** ^1^Jon M. Huntsman School of Business, Utah State University, Logan, UT, United States; ^2^Saïd Business School, University of Oxford, Oxford, United Kingdom; ^3^Department of Experimental Psychology, Katholieke Universiteit Leuven, Leuven, Belgium; ^4^Department of Experimental Psychology, Utrecht University, Utrecht, Netherlands

**Keywords:** perception, cognition, ecological rationality, psychophysics, biology, uncertainty, decision making, behavioral economics

## Abstract

In this paper we contrast bounded and ecological rationality with a proposed alternative, generative rationality. Ecological approaches to rationality build on the idea of humans as “intuitive statisticians” while we argue for a more generative conception of humans as “probing organisms.” We first highlight how ecological rationality’s focus on cues and statistics is problematic for two reasons: (a) the problem of cue salience, and (b) the problem of cue uncertainty. We highlight these problems by revisiting the statistical and cue-based logic that underlies ecological rationality, which originate from the *misapplication* of concepts in psychophysics (e.g., signal detection, just-noticeable-differences). We then work through the most popular experimental task in the ecological rationality literature—the city size task—to illustrate how psychophysical assumptions have informally been linked to ecological rationality. After highlighting these problems, we contrast ecological rationality with a proposed alternative, generative rationality. Generative rationality builds on biology—in contrast to ecological rationality’s focus on statistics. We argue that in uncertain environments cues are rarely given or available for statistical processing. Therefore we focus on the psychogenesis of awareness rather than psychophysics of cues. For any agent or organism, environments “teem” with indefinite cues, meanings and potential objects, the salience or relevance of which is scarcely obvious based on their statistical or physical properties. We focus on organism-specificity and the organism-directed probing that shapes awareness and perception. Cues in teeming environments are noticed when they serve as cues-for-something, requiring what might be called a “cue-to-clue” transformation. In this sense, awareness toward a cue or cues is actively “grown.” We thus argue that perception might more productively be seen as the *presentation* of cues and objects rather than their *representation*. This generative approach not only applies to relatively mundane organism (including human) interactions with their environments—as well as organism-object relationships and their embodied nature—but also has significant implications for understanding the emergence of novelty in economic settings. We conclude with a discussion of how our arguments link with—but modify—Herbert Simon’s popular “scissors” metaphor, as it applies to bounded rationality and its implications for decision making in uncertain, teeming environments.

## Introduction

Recent theories of bounded and ecological rationality focus on the structural and statistical properties of environments. Humans are seen as *intuitive statisticians* who process their surroundings by relying on a “statistical toolbox” of heuristics (Peterson and Beach, 1967; Gigerenzer and Murray, 1987; Cosmides and Tooby, 1996; Goldstein et al., 2001; Scheibehenne et al., 2013; Meder and Gigerenzer, 2014; Gigerenzer and Marewski, 2015; Gigerenzer, 2020).

Over the past decades, the concept of a *cue* has become foundational to this literature (for a review, see Gigerenzer and Gaissmaier, 2011; also see Gigerenzer and Goldstein, 1996; Karelaia and Hogarth, 2008; Marewski et al., 2010). Cues are essentially seen as data or “pieces of information in the environment” (Kozyreva and Hertwig, 2021, p. 1526). Cues represent the data and information that needs to be processed to attain rational judgments and outcomes (Gigerenzer, 2020; Hertwig et al., 2021). This focus on cues has lent itself to applying (or as we argue, misapplying) a whole host of assumptions and methods from psychophysics and statistics to understand and study rationality. The methods used to highlight the idea of humans as intuitive statisticians include various approaches such as random sampling, signal detection, stimulus thresholds, lens model statistics, just-noticeable-differences, Neyman–Pearson statistics, representative design, and Bayesian inference (e.g., Dhami et al., 2004; Hogarth, 2005; Pleskac, 2007; Karelaia and Hogarth, 2008; Hertwig and Pleskac, 2010; Todd and Gigerenzer, 2012; Luan et al., 2014; Gershman et al., 2015; Gigerenzer and Marewski, 2015; Feldman, 2017; Rahnev and Denison, 2018; Szollosi and Newell, 2020).

In this paper we argue for a generative approach to rationality, one that focuses on humans as probing organisms rather than intuitive statisticians. While the ecological rationality literature is strongly anchored on statistics, we build on biology. In the paper we first discuss two problems with the ecological rationality literature’s focus on cues and humans as intuitive statisticians: (a) the problem of cue salience, and (b) the problem of cue uncertainty. The emphasis on the physical and statistical aspects of cues—as data to be processed—misses the fact that the relevant cues may lack these qualities. The focus on statistically or physically measurable factors—concepts imported but misapplied from psychophysics: size, intensity, frequency, repetition and so forth (cf. Gigerenzer and Gaissmaier, 2011)—treats cues as predefined or given. In the paper we work through the most popular and frequently discussed experiment in ecological rationality—the city size task—and highlight how psychophysical intuition has been extended to the context of rationality in problematic fashion. We argue that “ready-made” conceptions of environments cannot deal with the question of how cues emerge in the first place, as illustrated by situations where relevant or critical cues are small, non-obvious or hidden.^1^ This problem is exacerbated in real-world “teeming” environments, which differ wildly from the environments used in experimental tasks. We revisit the foundations of these arguments—linking to early work by Fechner and others—and the problem of how one might “grow” a cue.

In response to existing work, we develop a generative alternative to rationality, an approach that addresses the aforementioned problems of cue noticeability, relevance and novelty. We argue that environments are organism-specific and that organism-directed search plays a critical role in shaping cue salience. In real-world situations and tasks—particularly in teeming environments—the relevant cues and environmental structure are rarely if ever predefined, given or obvious. Rather, cues are noticed when they serve as cues-for-something (Koenderink, 2011, 2012; cf. Chater et al., 2018)—that is, clues or evidence. In situations of judgment and rationality, noticing the relevant cues has more to do with organism-specific, generative factors rather than bottom-up statistical ones (like thresholds or signal detection). We discuss the need to understand what might be called the cue-to-clue transformation, that is, how organism-specific, top-down factors play a role in transforming “raw” optical structure and latent or dormant cues into clues-for-something. In essence, we provide an alternative theory of noticing—a generative approach to understanding salience, cue “growth” and detectability. We link these arguments to bounded rationality and decision making in uncertain environments, and conclude with a reconceptualization of Herbert Simon’s popular “scissors” metaphor.

## Ecological Rationality, Cues and Statistics: a Brief Review

The concept of an environmental *cue* is a foundational unit of analysis within the bounded and ecological rationality literatures (Gigerenzer and Goldstein, 1996; Karelaia and Hogarth, 2008; Luan et al., 2019; Kozyreva and Hertwig, 2021). These literatures build on the premise that environments, as Todd and Gigerenzer (2020, p. 15; also see Hertwig et al., 2021) recently summarize this argument,

“…can be characterized by distributions of cues and cue values (how many there are, what range of values they can take, etc.), cue validities (how often a cue indicates appropriate decisions), redundancies (inter-cue correlations), and discrimination rates (how often a particular cue distinguishes between alternatives, regardless of its accuracy).”^2^

The focus on cues—as information and data (Todd and Gigerenzer, 2007; Luan et al., 2011)—has enabled scholars to statistically measure and specify the properties and structure of environments. The literature argues that “ecological, or environmental, structures are *statistical* and other descriptive properties that reflect patterns of *information distribution* in an ecology” (Kozyreva and Hertwig, 2021, p. 13, emphasis added). By focusing on cues, scholars have essentially sought to dimensionalize and quantify environments in various ways, by measuring factors such as the number of cues, or their redundancy, addition, growth, distribution, ordering, correlation, integration, combination, weighting and so forth (for a review, see Gigerenzer and Goldstein, 2011; also see Hutchinson and Gigerenzer, 2005; Chater et al., 2018). Importantly, cues are seen as an *a priori*, statistical property of the environment (Hertwig et al., 2021; Kozyreva and Hertwig, 2021).^3^

Ecological rationality starts with the premise—given its roots in bounded rationality (Gigerenzer and Selten, 2001)—that humans are not able to omnisciently or exhaustively capture, process and compute environmental cues, due to human limitations in “computational capabilities” (Simon, 1990; also see Gigerenzer, 2000; Kahneman, 2003; Lieder and Griffiths, 2020). The ecological rationality literature thus builds on the bounded rationality literature which recognizes that exhaustive or perfect representation is not possible (Simon, 1956). Given the lack of time and computational power, humans face varied trade-offs, including the trade-offs between satisficing versus optimality, good enough versus best, and accuracy versus effort (e.g., Gigerenzer and Brighton, 2009).

Given that omniscient processing and rationality is not feasible, the ecological approach to rationality points to (and offers) varied statistical shortcuts for making rational decisions—a so-called “statistical toolbox” of heuristics. Humans are seen as intuitive statisticians who utilize this statistical toolbox to simplify the process of understanding their environments to make rational decisions (Gigerenzer, 1992; cf. Cosmides and Tooby, 2013). This approach begins with the idea that rationality is best achieved by first, as discussed above, understanding the statistical *structure* of environments (Gigerenzer and Gaissmaier, 2011). And this structure, then, needs to be matched with the right shortcut, or statistical tool and heuristic. In other words, the goal is to “[analyze] the information-processing mechanism of the heuristic, the information structures of the environment, and the match between the two” (Todd and Gigerenzer, 2012, p. 5). To illustrate, in some situations it’s rational for an agent to randomly sample environmental cues and thus attain a locally optimal choice (Dhami et al., 2004). That is, rather than needing to engage in exhaustive or complete sampling of environmental cues, data and information, scholars have pointed out how in many situations it’s rational to sample on a more delimited basis (Hertwig and Pleskac, 2010). The so-called “less-is-more” heuristic suggests that sampling on a more delimited basis can be just as efficient as “perfect” rationality, which wastes cognitive resources (e.g., Katsikopoulos et al., 2010). Heuristics, then, are said to allow organisms and humans to attend to and sample cues on a more delimited and less costly basis, attaining decisions that not only are good enough but perhaps even equivalent to omniscience or unbounded forms of rationality (Todd and Gigerenzer, 2000).

The ecological rationality literature has developed a growing, statistical toolbox of heuristics. This statistical toolbox now includes tools such as random sampling, signal detection, stimulus thresholds, lens model statistics, just-noticeable-difference, Neyman–Pearson statistics, representative design, and Bayesian inference (e.g., Dhami et al., 2004; Hogarth, 2005; Pleskac, 2007; Karelaia and Hogarth, 2008; Hertwig and Pleskac, 2010; Todd and Gigerenzer, 2012; Luan et al., 2014; Pleskac and Hertwig, 2014; Gershman et al., 2015; Gigerenzer and Marewski, 2015; Feldman, 2017; Rahnev and Denison, 2018; Szollosi and Newell, 2020). And these statistical tools can directly be mapped onto various named heuristics (see Todd and Gigerenzer, 2012). The overall focus on humans as “intuitive statisticians” has been a central pillar of this literature for a number of decades (see Gigerenzer, 1992; Cosmides and Tooby, 1996). And this idea of course is echoed in earlier work as well. For example, Peterson and Beach (1967, p. 43) argued “experiments that have compared human inferences with those of statistical man show that the normative model provides a good first approximation for a psychological theory of inference.” This conception of the human statistician has enthusiastically been endorsed in ongoing work (Meder and Gigerenzer, 2014, p. 130; Hertwig et al., 2018).

Before proceeding, we might note that ecological approaches explicitly argue that these statistical tools and heuristics are the result of long-run, evolutionary adaptations to changing environments. As put by Gigerenzer (2008, p. 20), “the adaptive toolbox is a Darwinian-inspired theory that conceives of the mind as a modular system that is composed of heuristics, their building blocks, and evolved capacities.” Ecological rationality sees the human mind as composed of varied evolved statistical modules, including modules like Bayesian inference, signal detection, and so forth (see Figure 1, Gigerenzer, 1992, p. 336). Ecological rationality builds on a broader program of research in evolutionary psychology, where “the brain is a computer…designed by natural selection”—and, “if you want to describe its operation in a way that captures its evolved function, you need to think of it as composed of programs that process information” (Cosmides and Tooby, 2013, p. 203). This emphasis on computation and statistical processing provides the ongoing foundation for the ecological rationality literature (Gigerenzer, 2020), as well as generalized models of cognition and rationality (e.g., Gershman et al., 2015; Lieder and Griffiths, 2020).

## Cues and Environments: Two Problems

While the notion of humans as intuitive statisticians—and the statistical toolbox of heuristics—has offered useful insights, this literature is overly-reliant on the assumption that environments can be statistically captured, or that the relevant cues can be predefined. As we will show, approaches that treat cues as given and environments as, essentially, “ready-made,” have not fully come to terms with where cues come from in the first place and the “teeming” nature of real decision environments. To illustrate these points, we discuss how ecological approaches to rationality suffer from two specific problems: (a) the problem of cue salience, and (b) the problem of cue uncertainty. We discuss these two problems by revisiting existing experimental work and by linking the foundations of ecological rationality to psychophysics. Thereafter we propose an alternative, “generative” approach to rationality.

Note that our criticisms here are *not* meant to offer a wholesale challenge to the contributions of the ecological rationality literature. Instead, our efforts might be seen as setting boundaries for the generality of ecological approaches that focus on cues and the idea of humans as “intuitive statisticians.” More importantly, our discussion of these problems is meant to provide a jumping-off point and rationale for developing an alternative approach to rationality, one that is focused on organism-specific and directed, generative factors which are essential for understanding rationality in uncertain environments.

### The Problem of Cue Salience

One way to recast ecological rationality is to point out how its underlying “theory of noticing” is focused on the quantitative or statistical properties of cues—factors such as the *amount, intensity and distribution* of cues (see Gigerenzer and Gaissmaier, 2011). This is perhaps most evident in the emphasis on “*stimulus detection* as intuitive statistics” (Gigerenzer, 1992). Stimulus detection of course implies knowing what in fact counts as a stimulus. Importantly, in the existing literature the specific mechanism of detecting the stimulus is focused on the amount or “size” of a particular cue. To put this informally, a predefined and given cue is perceived or recognized when there is “lots” of it.^4^ As recently summarized by Kozyreva and Hertwig (2021, p. 1531), “sample size itself becomes an important environmental structure.” In essence, the underlying theory of noticing—in the simplest of terms (though we add nuance below)—is that noticing is dependent on the proverbial loudness, amount or size of cues: factors that can be physically and statistically measured.

This focus on the statistical and physical aspects of cues—sometimes called inputs, stimuli or data (Gigerenzer, 2020)—builds on a long historical tradition in psychology. The foundations of this work were laid by scholars such as Ernst Weber and Gustav Fechner in psychophysics (Boring, 1942; Wixted, 2020). We revisit the central elements of this work. Doing so is important because these building blocks of psychophysics are the *de facto* foundation of the ecological rationality literature (e.g., Gigerenzer, 1992, 2020; Luan et al., 2011, 2014). In other words, roughly the same mechanisms of salience—the underlying theory of noticing—are employed in both literatures. This underlying foundation of signal detection, just-noticeable-differences and stimulus thresholds was essential for the early work in ecological rationality (Gigerenzer, 1991, 1992) and continues to be centerstage to this day (see Karelaia and Hogarth, 2008; Luan et al., 2011, 2019; Gigerenzer, 2020). However, we argue that these psychophysical foundations have been wrongly applied in the context of ecological rationality.

The goal of early work in psychophysics was to experimentally study if and when humans notice—and become aware of—a *given, prespecified* cue or stimulus (Boring, 1942). In the earliest formal experiments, Gustav Fechner introduced human subjects to a single stimulus—an auditory, haptic or visual one—and proceeded to see *when* the focal stimulus became salient. Fechner’s approach was to gradually, in small increments, increase the amount of the focal cue and then to see when subjects noticed and became aware of it. His underlying approach, as he put it in his classic *Elements of Psychophysics*, was to start from “zero” and then to essentially “grow” awareness toward particular physical cues and stimuli. As Fechner (1860, p. 58) put it, stimuli “might be seen as incrementally grown from zero” (in the original German: “aus positiven Zuwüchsen von Null an erwachsen angesehen werden” – our translation).

This early work in psychophysics sought to provide a scientific basis for psychology, a way to rigorously quantify and statistically measure physical stimuli and cues in environments. One aim of this approach was to make psychology more like the hard sciences, like physics, where the amounts and quantities of cues or stimuli served the equivalent of mass and force. Awareness was essentially seen as a function of the metaphorical mass of something—the amount, intensity and frequency of the cue. Fechner’s work became the basis of signal detection theory, a ubiquitously important theory that offered a statistical and quantitative basis for how increased intensities or amounts of stimuli were the central variable of interest for understanding perception and awareness (see Link, 1994; Wixted, 2020). This work also became the basis of theories of signal detectability (TannerJr., and Swets, 1954; also see Peterson and Birdsall, 1953), which have also had a strong influence on ecological rationality (e.g., Gigerenzer, 2000).^5^

This logic continues to pervade behavioral economics more broadly, where salience is seen as the “the property of a stimulus that draws attention bottom up” (Bordalo et al., 2021, p. 6). Or as put by Kahneman (2003, p. 1453), “the impressions that become accessible in any particular situation are mainly determined, of course, by the *actual* properties of the object of judgment,” and “physical salience [of objects and environments] determines accessibility.” Thus the emphasis is on predefined cues and whether humans appropriately process them based on their physical and statistical characteristics.

Early work in psychophysics—specifically the work of Ernst Weber—also looked at when humans noticed *comparative* differences between two cues or stimuli (Weber, 1834; for a review, see Boring, 1942; Algom, 2021). Here the premise again was to start from zero: a “zero” difference between two cues (e.g., optical stimuli, lifted weights, or sounds), and then to incrementally increase the brightness, weight, or loudness of one of the stimuli to see when the comparative difference was noticed. As summarized by Gigerenzer (1992, p. 339), “detection occurs only if the effect a stimulus has on the nervous system exceeds a certain threshold value, the ‘absolute threshold.’ Detecting a difference (discrimination) between two stimuli occurs if the excitation from one exceeds that of the other by an amount greater than a ‘differential threshold’.” This logic provides much of the foundational intuition behind ecological rationality. Scholars have debated whether absolute or relative differences matter more within the context of judgment and decision making (e.g., Hau et al., 2010; Hertwig and Pleskac, 2010). But the underlying foundations of Weber’s pioneering work—concepts such as just-noticeable-differences—continue to be center stage in ecological rationality literature (e.g., Pleskac and Busemeyer, 2010; Luan et al., 2014; Gigerenzer, 2020).

Importantly, Weber and Fechner’s work on stimulus comparison and difference detection had been extended into the domain of judgment and decision making earlier, by scholars like Thurstone (1927). Thurstone developed his so-called laws of comparative judgment and discrimination, and these Thurstonian notions were in turn further extended by decision theorist Duncan Luce into axioms of choice and decision making, with a strong focus on the representation of signals and environments (Luce, 1963, 1977; also see Dawes and Corrigan, 1974). This work is also central to ecological rationality, particularly arguments about the representational nature of perception and rationality (e.g., Gigerenzer, 1991; Juslin and Olsson, 1997; Luan et al., 2014). But as we will discuss, these psychophysical foundations have been misapplied by the ecological rationality literature.

Now, these psychophysical foundations are clearly important for understanding certain aspects of perception.^6^ However, the central question here is whether the underlying statistical architecture of psychophysics—focused on noticing a stimulus as a function of its “amount” (such as frequency, intensity, size)—is sufficiently general for handling varied questions and situations of rationality. For example, how might we account for situations where the relevant cues have *none* of the traditional statistical or physical characteristics of salience? Also, the underlying logic psychophysics was to introduce *one* stimulus, and to identify when it was salient (based on amount), or to compare the relative salience between two stimuli (from a baseline of zero). But most environmental settings “teem” with indefinite cues and stimuli. As we highlight below, the focus on the amount—whether absolute or relative—does not generalize to situations where amounts simply are not relevant. A more central question—particularly in environments that teem with indefinite cues and stimuli—is how one might become aware of the *relevant* cues, amongst varied potential distractions and noise. The logic of incrementally growing or increasing the intensity of a given cue—or comparing two cues—does not translate to these types of settings.

The default starting point or initial condition of psychophysics might, in effect, be seen as a proverbial dark or silent room, where the intensity of a focal stimulus is gradually increased, dialed up and “grown”—to establish threshold levels of awareness or signal detection. While this of course is important (and certainly relevant for situations of visual or auditory impairment), and allows for scientifically clean and controlled conditions for explaining a highly particular form of awareness (when organisms “notice” something, or do not), it scarcely mimics many of the complex situations and teeming environments that humans and other organisms encounter and find themselves in. The idealized starting point of a metaphorical dark or silent room of psychophysics might instead be replaced by a different metaphor. A better default metaphor might be captured by a human standing midday at Times Square in New York, encountering indefinite visual and auditory stimuli, bombarded by innumerable sounds and sights. This real-life “Wimmelbild” better captures the problem faced by a decision maker in an uncertain environment. This teeming visual scene, like any other, is full of “signals” and “affordances” (Krebs and Dawkins, 1984; Koenderink, 2012) which cannot be accounted for by any kind of generic focus on the physical or statistical aspects of the scene.^7^

Now, while it has not meaningfully been integrated into the ecological rationality literature, there is of course a larger literature in the domain of perception that has wrestled with how humans process cues and information in “busy,” multisensory environments. This literature has focused on such questions as how we might bind, combine or separate particular cues and sensory inputs in visual scenes and environments (e.g., Treisman and Gelade, 1980; Landy et al., 1995; Noppeney, 2021; Wolfe, 2021). While this literature is important, it also builds on the aforementioned psychophysical premise where cues and features are given, and salience is driven by physical or statistical factors, specifically the *relationships* amongst the cues (for example: the spatial distance of cues, cue similarity or difference). This research presents experimental subjects with varied arrays of visual cues or scenes and looks at how and whether humans process them veridically. While this work certainly has its place (particularly in contexts of establishing sensory deficiencies), it builds on an “all-seeing” conception of perception (Koenderink, 2014; Hoffman et al., 2015; Felin et al., 2017). Thus we think different perceptual foundations are needed for understanding judgment and rationality in uncertain environments (cf. Chater et al., 2018).

The so-called “cocktail party effect” or cocktail party problem (Cherry, 1953; Shinn-Cunningham, 2008) offers a somewhat better instantiation of the types of teeming environments encountered by organisms, humans included. Despite its obvious relevance, the cocktail problem surprisingly has not been cited or addressed in the ecological rationality literature. The cocktail problem is the very relatable problem of how one focuses on a particular conversation or auditory stimulus in a noise-filled environment filled with distractions. In this type of teeming situation, cue salience is *not* given by any form of statistical aspects of the cues themselves (e.g., how loud a stimulus is). We might of course highlight which cues are, in a relative psychophysical sense louder and thus seemingly more salient than others. But here the question is rather about selecting and picking out a relevant conversation or cue. In these situations, salience is given by deliberate, top-down mechanisms on the part of subjects. This literature thus focuses on factors such as motivation and interest as drivers of cue salience, in an environment filled with other cues and distractions. Another parallel is the literature focused on “motivated perception,” “motivated seeing” and “wishful seeing” (Bruner and Goodman, 1947; Balcetis and Dunning, 2006; Leong et al., 2019). However, these literatures have largely focused on the biased or self-delusional nature of hoping or wanting to see and find something (a form of confirmation bias), rather than rationality-related considerations and concerns.

In all, we might summarize ecological rationality as follows. Ecological rationality treats the world as a dataset to be processed, where the cues and data are given. The role of the human, as intuitive statistician, is to efficiently process these cues using heuristics and associated statistical tools. However, what is lost in these abstractions is the often messy and critical process of deciding what represents a cue in the first place, or how a potentially “small” or hidden (but relevant) cue might somehow be identified or detected. As we discuss (see section below: “Humans as Probing Organisms”), in many situations of judgment and decision making, the relevant cues are scarcely obvious. And importantly, critical cues often do not have any of the traditional psychophysical characteristics of being loud, intense or large. Thus some alternative mechanisms for generating salience are needed.

### The Problem of Cue Uncertainty

While the literature on ecological rationality emphasizes that it is squarely focused on decision making in the context of *uncertainty*, yet the most common experiments and tasks are relatively straightforward, even mundane. But as we illustrate next, it’s hard to know how the key experiments and examples of ecological rationality actually generalize to novel situations and real-world environments that teem with more radical forms of uncertainty.

To illustrate this problem, consider the most popular experiment and example used by scholars of ecological rationality, the city size comparison task. The city size task is a useful example as it is the focal experiment of the most highly cited academic article in the ecological rationality literature (Gigerenzer and Goldstein, 1996) and also extensively discussed in highly cited books (e.g., Gigerenzer and Todd, 1999). Furthermore, variants of the city size experiment have been done across numerous different contexts over the past three decades, published in various top psychology and cognitive science outlets (e.g., Gigerenzer et al., 1991; Goldstein and Gigerenzer, 2002; Chater et al., 2003; Schooler and Hertwig, 2005; Pohl, 2006; Richter and Späth, 2006; Dougherty et al., 2008; Gigerenzer and Brighton, 2009; Marewski et al., 2010; Hoffrage, 2011; Pachur et al., 2011; Heck and Erdfelder, 2017; Filevich et al., 2019). The city size experiment has also been highlighted as an example of different heuristics, including the recognition heuristic, as well as the less-is-more, tally, and take-the-best heuristics (Goldstein and Gigerenzer, 2008). In all, the city size experiment appears to be the most popular experiment in the ecological rationality literature. Thus it serves as a useful example for us to make our point, namely, that it’s hard to see how the arguments about ecological rationality generalize to decision situations and environments that actually feature uncertainty. Furthermore, the city size experiment offers a practical example of how the basic logic of psychophysics—and the associated statistical toolbox—has been imported and translated into the domain of ecological rationality.

In a prototypical city size experiment, subjects are presented with pairs of cities and asked to estimate which of the two cities has a larger population. Subjects might be asked whether, say, Milan versus Modena has more inhabitants (Volz et al., 2006)—or whether Hamburg versus Cologne (Gigerenzer and Goldstein, 1996), Detroit versus Milwaukee (Neth and Gigerenzer, 2015) or San Diego versus San Antonio (Chase et al., 1998) has a larger population. In some experiments subjects are asked to compare cities in their country of residence—or sometimes in a foreign country, or both (see Chater et al., 2003; Pohl, 2006; Richter and Späth, 2006; Gigerenzer and Brighton, 2009; Marewski et al., 2010). Though there are any number of variants to the experiment, the most basic version of the experiment is one where subjects are given a city pairing and simply asked to guess or pick the more populous city. The upshot is that, in a relatively high percentage of instances (higher than chance), the guesses and picks of experimental subjects turn out to be correct.

The popular city size experiment is said to be an example of—amongst other things—the “recognition heuristic.” The recognition heuristic is relatively intuitive and simple, defined as follows: “If one of two objects is recognized and the other is not, then infer that the recognized object has the higher value with respect to the criterion” (Schooler and Hertwig, 2005, pp. 611–612; Volz and Gigerenzer, 2012, p. 3; first defined in these particular terms in Goldstein and Gigerenzer, 2002, p. 76). To put the recognition heuristic in the context of the city size experiment, the idea is that while experimental subjects might not actually know which (say German) city has a larger population, the process of recognizing the name of one of the comparison cities can serve as a useful shortcut or heuristic for making the correct choice. If an American experimental subject is asked whether, say, the city of Munich or Cologne has a larger population, they might draw on other cues and information to enable them to pick the larger city. For example, an experimental subject might have visited Germany and thus be more likely to have flown into Munich, since it is Germany’s second largest airport for international flights. Or an experimental subject might be aware of other facts about Munich—for example, that Oktoberfest is based in Munich. Or they might be aware of the popular German soccer team Bayern Munich.

The idea behind the recognition heuristic is that these cues or “ancillary” bits of information can serve as additional information for recognizing Munich, and therefore arriving at the correct decision about its size relative to Cologne. In some of the experiments, subjects are given some form of additional or related cues, or primed to focus on certain ones (e.g., Gigerenzer and Goldstein, 1996), and in others they are simply given the pair-wise city comparisons and asked to choose the city with the larger population (Pohl, 2006; Todd and Gigerenzer, 2012). But the key point that scholars of ecological rationality hope to make with the city size and related experiments is that a subject’s informational recall and memory essentially serve as a shortcut to amass and tally cues to increase the probability that they arrive at the correct decision. While the city size experiment has largely been used to highlight the recognition heuristic, the same experiment has also been used as an example of a host of other heuristics, including heuristics like take-the-best, less-is-more and tally (weighted and unweighted) (see Todd and Gigerenzer, 2012).

The city size experiment and its variants are highly informative as they show how scholars in ecological rationality essentially borrow and translate the logic of psychophysics and cues into the context of heuristics and decision making (Gigerenzer and Gaissmaier, 2011). Cues are treated synonymously with varied, discrete bits of information about the cities. These cues, then, are the metaphorical equivalent of psychophysical “growing a cue from zero”—where information accumulates toward the correct judgment. Again, if a subject is given the task of deciding whether Munich versus Cologne has a larger population, simply knowing about Bayern Munich represents one cue or bit of information that favors its selection. And knowing that Munich hosts the Oktoberfest might serve as another, and so forth. This allows scholars to apply psychophysics-type intuition where the cues are *tallied, weighted, sequenced, and ordered* (and so forth) in different ways (cf. Karelaia and Hogarth, 2008). In other instances, knowing (or being given) some additional facts about a given city is treated in probabilistic fashion (called “probabilistic mental models”), where increased information about a particular city increases one’s confidence that it will have a larger population (Gigerenzer et al., 1991).

The full logic of the city size argument, linking psychophysical cues with bounded and ecological rationality, is simulated and worked out by Gigerenzer and Goldstein (1996) in their article titled “Reasoning the fast and frugal way: Models of bounded rationality.” They create a computer simulation that features a set of competing heuristics (or algorithms) for estimating the population size of 83 German cities (i.e., all cities in Germany with more than 100,000 inhabitants). The set of cues used to engage in this task includes nine binary (yes/no) bits of information about each city—for example, whether the city has a soccer team in the Bundesliga, whether the city has a university, or whether the city has an intercity train. This same logic has been applied to many other decision environments (for a summary of 27 different ones, see Todd and Gigerenzer, 2012, pp. 203–206). And these findings have not just been simulated, but variants of this approach have been studied with experimental subjects (Goldstein and Gigerenzer, 2002; Dieckmann and Rieskamp, 2007).

Ecological rationality’s focus on the city size experiment—and similar tasks—tells us a lot about the approach. It reduces judgment and decision making to a type of signal detection and statistical processing. This is further evident in, for example, applications of the cue-based logic of Brunswik to the city size problem (e.g., Gigerenzer et al., 1991; Hoffrage and Hertwig, 2006). The idea of Bayesian inference is also featured prominently in the city size task and heuristics literature more broadly (Chase et al., 1998; Goldstein and Gigerenzer, 2002; Martignon and Hoffrage, 2002), given the obvious links to signal detection. The idea is that humans don’t exhaustively process information, but they use a statistical toolbox—sampling to make their choices. This logic has been applied and extended to many other tasks of comparison and estimation, such as mammal lifespans, car accident rates, the number of species on Galapagos Islands, homelessness, and car mileage (see Todd and Gigerenzer, 2012, pp. 203–206; for a metareview, see Karelaia and Hogarth, 2008).

Now, in principle there is no problem with highlighting how humans might use varied cognitive shortcuts and tricks to enable them to arrive at correct answers about such questions as which city has a larger population, or who (say) won a particular historical match at Wimbledon (Todd and Gigerenzer, 2007). It seems very plausible that humans use shortcuts like this, using ancillary cues and information as a guide. The recognition and associated heuristics undoubtedly can prove useful in the types of situations and experiments constructed by the experimenter.

But our concern is that the most popular examples and experiments of ecological rationality—like the city size experiment—seem to scarcely generalize to other settings, situations and tasks where the relevant cues are not given, and where the right answer simply cannot be looked up. This is a problem, because the focus of ecological rationality is supposed to explicitly be on “situations of uncertainty where an optimal solution is unknown” (Gigerenzer, 2020, p. 1362). The city size and related experiments scarcely are an example of an uncertain situation. While these experiments are highly prominent in the ecological rationality literature, it’s extremely hard to see how they might tell us something meaningful about judgment and decisions in truly uncertain, teeming environments.

## Humans as Probing Organisms: a Generative Approach

Next we develop an alternative, generative approach to rationality, in response to some of the aforementioned problems we have identified with ecological rationality. Our generative alternative argues that humans might best be seen as probing organisms rather than intuitive statisticians. While ecological rationality builds on statistics, we build on and extend biological arguments and develop a more generative form of rationality.

We should note that in juxtaposing the aforementioned discussion of humans as intuitive statisticians with our generative alternative, we certainly do not want to offer a wholesale challenge to existing, ecological arguments. The two approaches have their respective benefits, *depending on the task or problem at hand*. We recognize that the logic of intuitive statistics can be applicable to certain settings and for specific types of tasks, where the relevant cues are given and varied forms of statistical processing indeed might be useful. But our proposed alternative might be seen as establishing some much-needed boundaries and contingencies for ecological rationality and related arguments. And more importantly, we hope to highlight how our generative alternative offers a more viable (though admittedly tentative) and biologically grounded option for judgment and decision making, especially in uncertain environments.

### Organism-Specific, Teeming Environments

Rather than seeking to first, *a priori*, dimensionalize or quantify environments—based on the redundancy, sample size or the variability of cues (see Gigerenzer and Gaissmaier, 2011, p. 457)—the generative approach starts with the premise that environments are organism-specific. As put by Goldstein (1963, p. 88), “environment first arises from the world only when there is an ordered organism.” From our perspective there is no *a priori* environment or environmental structure to be accounted for in the first place—whether statistically or otherwise—without first understanding the organism in question (cf. Schrödinger, 1944; Riedl, 1984; Uexküll, 2010). What an organism is aware of, what becomes salient to it, and what it sees, is organism-dependent. While this might sound like an obvious statement, this organism-dependence—including its downstream consequences for rationality—has not been recognized, as we will illustrate.^8^

Organism-specificity means that an organism’s physiology and nature are central to understanding what its environment is (Tinbergen, 1963; Uexküll, 2010). As put by the biologist Uexküll (2010, p. 117), each organism exists in its own surroundings (what he called “Umwelt”), where certain species-specific things are visible and salient to it: “every animal is surrounded with different things, the dog is surrounded by dog things and the dragonfly is surrounded by dragonfly things.” At the most basic level, organism-specificity means that organism perception is given by what the organism’s visual and sensory organs enable it to see. Sensory organs provide the enabling and constraining mechanism for what the organism can see in its environment, allowing the organism to perceive certain things it encounters, but not others. Certain stimuli, cues, colors, objects are inherently salient to particular organisms. For example, humans can see the visual electromagnetic spectrum between 700 and 400 nm, while bees can detect light between 600 and 300 nm, which includes ultraviolet light (between 400 and 300 nm – not visible to the “naked” eye). Visual scenes and environments therefore look fundamentally different to different species (Cronin et al., 2014; Marshall and Arikawa, 2014). Importantly, this visual heterogeneity applies not only to colors and the electromagnetic spectrum but also to the set of *objects* that are salient and evident to a given species.^9^

As Caves et al. (2019) recently emphasize, treating environments the same across species is a common problem in the sciences, creating significant biases in how we talk about perception, judgment and environments. By treating the environment in homogeneous fashion, we succumb to faulty assumptions like assuming that animals are “doing the math” (or behaving “as if” they did the math: cf. Gigerenzer, 2021), or assuming that different organisms segment cues and stimuli in the very same ways that humans do. These biases have extended into the judgment and decision making literature where scholars have, for example, compared bee cognition with human cognition, suggesting that humans in many instances are less rational than certain animals (Stanovich, 2013). Or in other instances scholars have compared human perception with the “biased” and non-veridical perception of, say, a house fly (Marr, 1982, p. 34; cf. Hoffman et al., 2015, p. 1481). From our perspective, there is no “biased” nor veridical perception of an environment, where one view somehow is more veridical or more/less biased than another. These types of claims succumb to an “all-seeing” view of perception, a view that remains pervasive even though it is untenable (Koenderink, 2014; also see Felin et al., 2017). The problem is that we assume that disparate organisms perceive, or should perceive, the same cues and stimuli in the same way in a given environment—that there is a form of global optimality or omniscience. But this is scarcely the case. Environments are as heterogeneous as the organisms in them.

Now, so far we’ve emphasized visual heterogeneity across species, highlighting different forms of perception and the indefinite, teeming nature of any environment. But what about visual heterogeneity “within” species? Or put differently, what does any given organism, a human included, see at any particular moment? This moment-by-moment visual heterogeneity *within* a given species or organism is critical for our arguments, as visual metaphors and arguments are the foundation of much of the rationality literature (see Simon, 1956; Kahneman, 2003; Chater et al., 2018). The critical question is, if visual scenes and environments teem with potential objects and things—far beyond any ability to capture them all—then what is salient and visible to an organism at any given moment? Here the answer is not about what a given organism *can* see (as enabled by the organism’s sensory organs, discussed above), nor is it about any form of *ex ante* physical salience (as suggested by psychophysics and ecological rationality). Instead, our focus is on what an organism *might* become aware of at any given moment, amongst indefinite environmental possibilities.

The biologist Uexküll’s (2010) notion of a Suchbild (German for “search image”) offers a powerful way to think about moment-by-moment awareness. It suggests that, at the simplest level, organism perception is directed toward what it is looking for, whether it be foraging for food or looking for shelter. This Suchbild might be innate (like in the case of the frog looking for flies to eat) or cognitive (in the case of a humans, say, looking for their car keys). Salience is created by the image that the organism has in mind, the object or thing it is searching for (also see Tønnessen, 2018). Organisms fixate on certain visual features or objects—features and objects that essentially serve as the “answers” to their queries (cf. Felin and Kauffman, 2021). What is seen in the environment are the plausible answers or solutions to the organism’s search image. For example, when hunting and foraging for crickets, frogs are highly attuned to movement, perceiving motion (of a certain type) rather than perceiving the cricket itself (Ewert, 2004).

In the context of human perception, search images can be seen as a form of question-answer probing that guides visual awareness in our everyday life (Koenderink, 2012; Felin et al., 2017). For example, if I have lost my house keys, I scan my surroundings with a key search image in mind, looking for objects or stimuli that have key-like features. The search image allows me to ignore any number of other items and objects in my surroundings—even ones with psychophysically salient characteristics (like size)—and to focus on the task of finding my keys. Visual salience, then, is given by what I am looking *for*, offering a simplistic example of the intentional nature of perception.

Notice that this perspective suggests that perception is a form of active *presentation* rather than representation. That is, the organism plays a critical role in actively presenting certain stimuli or objects, rather than representing them (or the environment more broadly). As put by Brentano (1982/1985, pp. 78–79; also see Albertazzi, 2015), “by presentation I do not mean what is presented, but rather *the act* of presentation.”^10^ The sought-after object becomes salient, presenting itself to us through the process of active probing and search by the organism.

Our key point here is that *visual search is not just organism-specific but also task-, problem-, and object-specific.* That is, our moment-by-moment awareness happens in generative fashion and is structured by what we are looking for and “doing”—or asked to do—at any given moment.^11^ This generative and presentational lens on perception means that any appeals to notions of human perceptual “blindness” or bias—a common point of emphasis in the rationality literature (see Kahneman, 2011; Felin et al., 2017)—simply do not make any sense. This fundamentally changes how scholars of rationality should think about perception, particularly as perceptual and psychophysical arguments are at the very heart of rationality (Kahneman, 2003; for a review, see Chater et al., 2018). For example, Kahneman (2011, pp. 23–24) extends the core argument of the inattentional blindness literature (see Simons and Chabris, 1999) into the domain of judgment and rationality and argues that humans are “blind to the obvious.” But the reason humans “miss” things in their visual scenes—things that should be obvious (based on the logic of psychophysics)—is not because they are blind, but rather because they are engaged in tasks which direct their awareness toward other things (Felin et al., 2019). This points toward a “presentational” view of perception, where what presents itself are the cues or objects that we are looking for (or asked to look for), rather than a representational view that focuses on those cues or objects that have certain (*a priori*) psychophysical features or characteristics [what Kahneman (2003) calls “natural assessments” such as the size, distance or loudness of cues and objects].

A better way, then, to think about the organism-environment relationship—so fundamental to the bounded rationality literature (Simon, 1956, 1990; Gigerenzer and Gaissmaier, 2011)—might be to speak of a more fine-grained organism-object relationship instead. That is, moment-by-moment organism awareness is about specific objects that are situation- or task-relevant. The broader notion or word “environment” thus unwittingly creates a black box that needs to be unpacked. Awareness is *about* something specific in the environment (Brentano, 1982/1985; also see Brentano, 1995/1874), rather than about the environment as a whole. Psychophysical efforts seek to understand environments by treating them like data, pixels and dots—cues and statistical properties—and therefore miss this type of specificity and the indefinite potential objects that might be salient. To offer a simple metaphor, psychophysical and bottom-up approaches to environments treat it like an urn of cues and information, one that cannot exhaustively be sampled due to costs or computational limitations (Ellsberg, 1961; Edwards et al., 1963; see Brandstätter et al., 2006; Gigerenzer, 2021). The environment might be represented with an urn of, say, 10,000 red and black balls. And truth is then represented by a full knowledge of the relative proportion of the two different colors. Our task might be to somehow estimate this truth by sampling from the urn on a more limited basis, in heuristic fashion, given the costs associated with counting all of the balls. This urn-like conception of the environment allows ecological rationality to presume a quantifiable reality, matching heuristic and statistical techniques with that reality, and to compare varied heuristic techniques against an omniscient ideal. This type of simplification, of treating the environment like an urn (or set of cues and data points), has enabled the literature to focus on various statistical and probabilistic approaches to understanding environments (cf. Savage, 1950).

However, this urn-like, atomistic treatment and idea of sampling environments reduces environments to bottom-up cues and data. This is the metaphorical equivalent of assuming that one might understand a painting by adding up its constituent “dots” or pigments of color. To briefly extend the metaphor, consider Seurat’s painting *La Grande Jatte*, which consists of an estimated 220,000 dots (Goldstein, 2019). The problem is that no form of bottom–up sampling or quantification of these dots will communicate the same information as the top-down reading of the painting. The only thing we might learn from sampling the dots is how much of each color was used in the painting, but little else. But this is precisely how environments are metaphorically treated by ecological rationality (and literatures on scene statistics). This type of statistical analysis tells us nothing about the individual objects or subject-matter of the painting itself.^12^ The key point here is that: a bottom–up conception of environments doesn’t translate or scale to the real world in any meaningful way, except in limited circumstances.

Rather than speak of the broad organism-environment relationship, our focus is on the situation-relevant objects or cues within it. Perception is necessarily directed toward some object—for example, something we might be looking for—rather than the environment as a whole (or some disaggregated notion of the environment). Perceiving is *about* and *for* something specific, an object the organism is interested in. To offer an example, consider the work of Yarbus (1967). It offers a powerful example of how the search-for-something—like an answer to a question—shapes what presents itself and becomes salient and visible. Yarbus studied what he called the “perception of complex objects,” specifically by tracking the eye movements of experimental subjects, in an attempt to understand what humans perceive when encountering a teeming visual scene with disparate stimuli. For example, he tracked the eye movements of subjects viewing the artist Ilya Repin’s painting *The Unexpected Visitor.* Yarbus highlighted how a battery of prompts and questions that he posed shaped the stimuli and objects that were salient to experimental subjects. For example, he asked subjects to “estimate the material circumstances of the family in the picture,” or to “give the ages of the people,” or to “surmise what the family had been doing before the arrival of the unexpected visitor,” or to “estimate how long the unexpected visitor had been away from the family.” The upshot of this work is that it highlights how questions provide a type of search image for which answers are sought in visual scenes, presenting and creating salience for certain objects, cues and things at the “expense” of other things.

Notice how there is no single question that can somehow elicit all the feasible cues, objects and stimuli from a visual scene, whether we’re talking about Repin’s painting *Unexpected Visitor* or any other scene or environment. A generic prompt or request to simply “observe” or “describe the scene” might of course yield varied answers about the number of people in the picture, perhaps their ages, and so forth (or perhaps “typical” foci in human perception, like faces). But there’s no way to meaningfully exhaust visual scenes and environments. While some fields of psychology and cognitive science insist that this is possible, we argue that this simply is not the case (for a debate and discussion, see Chater et al., 2018). And importantly, as the Yarbus example highlights, there’s no way to speak of any form of psychophysical salience independent of the top-down questions and prompts that direct awareness. The salient things don’t inherently “shout” their importance, as assumed by psychophysics. Object obviousness is driven by the questions, interests or tasks specified by the organism or agent in question (Koenderink, 2012).

This underlying generative logic, as we discuss next, suggests a rather significant shift in how we think about perception, with important implications for the judgment, rationality and decision making literatures as well. While it might seem obvious that, say, questions direct awareness and salience, this logic remains radically under-appreciated and is counter to the key drivers of salience from the perspective of ecological rationality, where salience is said to be given by cue characteristics, environmental structure and statistics. And while there are mentions of “top-down” perception in the bounded and ecological rationality literatures, the focus remains on the perception of *predefined* cues. Thus we next revisit the idea of “growing” awareness and cues, and we highlight how dormant cues—not readily evident or obvious—might be identified and transformed into evidence or put differently, clues-for-something.

### Growing Awareness Toward (Relevant) Cues

As discussed above, psychophysics “grows” awareness toward cues based on their statistical or physical characteristics, such as intensity, frequency or size (Fechner, 1860). In its simplest form, the experimentalist essentially increases or “dials up” a specific stimulus, until awareness is reached. The focus on the amount-of-something as the critical ingredient (or mechanism) of perceptual salience is also the background logic behind “stimulus detection as intuitive statistics” (Gigerenzer, 1992), and the basis of the ongoing extensions of the logic of signal detection and size (whether sample or cue size) into the domain of ecological rationality (Gigerenzer, 2020; Kozyreva and Hertwig, 2021). To summarize (and oversimplify): psychophysics-based approaches argue that cue detectability is a function of how loud, big or intense a cue or stimulus is.

But what about situations where a critical cue has none of these salience-generating physical characteristics or statistical properties? What is the mechanism of salience in these situations? How might we detect something that is quiet, small and scarcely obvious but nonetheless highly relevant? Put differently, how is something that is hidden—or barely detectable—nonetheless detected? Is there a way of amplifying or “growing” awareness toward these types of cues? We address these questions next.

Our emphasis is specifically on the *psychogenesis* of awareness, rather than the psychophysics of perception and attention—a critical distinction (Koenderink, 2012, 2018; Felin et al., 2017). We essentially propose to offer an alternative, generative way of “growing” awareness toward a cue or “clue.”^13^ That is, rather than focusing on the intensity or size of a cue to enable its detection, we point to organism-specific, top-down mechanisms of detection. We point out how humans might become aware of “small”—seemingly non-obvious and undetectable—cues even when they have none of the traditional characteristics of salience.

Our approach to growing awareness toward a specific cue might best be introduced by an informal example. Consider Arthur Conan Doyle’s fictional detective story *The Adventure of Silver Blaze.* The story features a brief but informative bit of dialogue between the Scotland Yard detective and Sherlock Holmes:

*Scotland Yard detective*

Is there any other point to which you would wish to draw my attention?

*Sherlock Holmes*

To the curious incident of the dog in the night-time.

*Scotland Yard detective*

The dog did nothing in the night-time.

*Sherlock Holmes*

That was the curious incident.

The story describes a situation where the protagonists—a Scotland Yard detective and Sherlock Holmes—are engaging in an effort to identify the perpetrator of a crime. The investigators encounter and seek to systematically canvas an environment with innumerable cues and potential clues: people and their motives, a crime scene with innumerable objects (some visible, some not)—any number of *in situ* and *ex situ* variables that may or may not be relevant for solving the case. In short, the environment teems with indefinite, possible and dormant cues and potential clues.

The problem of course is that *anything* could be relevant: the fact that a door or window was left open (or not), the fact that some object is present (or missing) in a particular room, the fact that a chair is two versus three meters from a door, or that focal building in question is 120 miles from London, or that there is (or isn’t) a cigar butt on the ground, etc. In short, it’s impossible to know what might be relevant. Furthermore, the key clue or piece of information might be small and scarcely obvious. There’s no computational or statistical procedure for processing the scene. And important for our arguments, there is no *a priori* environmental structure that we might speak of.

The reason we highlight the above dialogue between Sherlock and the Scotland Yard detective is because it highlights a critical, generalizable point. Namely, one of the critical cues in this particular case (evident in the dialogue)—the dog that *didn’t* bark—has *no* physical or statistical properties whatsoever: it is not loud or large, it is not repeated, nor obvious in any meaningful way. There is no way to argue for psychophysical salience nor to point to some form of *a priori* representation. The example of course is fictional. But it nicely illustrates how a relevant cue might not meet any of the traditional characteristics of cue salience or detectability, as specified by psychophysics or ecological rationality. Rather, here we have a situation where the *lack* of an auditory sound—a dog *not* barking—is identified as curious and critical, providing vital information about the crime (in this case, the dog didn’t bark and therefore someone familiar with the dog was present at the crime scene).

The point we want to make is that cues do not say or mean anything by themselves. Just like in science, cues and data are meaningless without a theory or some alternative top-down factor, like a hypothesis, question or conjecture.^14^ The problem in science is that, as put by physical chemist Polanyi (1957, p. 31), “things are not labeled ‘evidence’ in nature” (for a recent discussion see Felin et al., 2021). Similarly, environmental cues don’t come with labels that say “this is relevant or important” or “this is evidence.” Cues—clues for something—are not inherently obvious. Furthermore, the size or amount of cues or samples cannot be equated with relevance or importance either. There is no “scene statistics” for resolving Sherlock’s case, just as there are no general statistics for processing visual scenes and environments (Koenderink, 2012). Cues are simply raw material and dormant data, until they are met with a probing organism and the right question. In this sense, cues are *made* visible rather than being inherently visible. Some form of top-down mechanism is needed to generate or grow awareness toward cues, to engage in what might be called a “cue-to-clue transformation.”^15^

Related to this transformation, it’s interesting to note that in Simon’s (1956) pathbreaking paper—“Rational choice and the structure of the environment”—he uses the word “clue” a number of times (while “cues” are the emphasis in the ecological rationality literature: Gigerenzer and Gaissmaier, 2011). Most of the instances of the word “clue” in Simon’s article are used in a relatively traditional psychophysical sense, where clues are perceptually seen based on their vicinity (“an organism’s vision permits it to see a circular portion”—Simon, 1956, p. 130; cf. Kahneman, 2003). But at the end of the article the word “clue” is parenthetically used in a more investigative and anticipatory sense. Specifically, Simon (1956, p. 136, *emphasis added*) discusses how an organism might search an environment randomly, or alternatively, on the basis of “clues in the environment (either the actual visibility of need-satisfying points or *anticipatory clues*).” It’s Simon’s parenthetical remark about “*anticipatory* clues” that finds some resonance with our discussion of generative rationality here. That is, an organism’s ability to recognize and see something as a clue might be *independent* of proximity (visual proximity or distance being the key mechanism of salience for the bounded rationality literature) or other psychophysical measures of salience (such as size). In other words, cue salience can also emerge independent of distance or independent of other physical characteristics. Our approach here can be seen as an effort to develop the organism-specific factors that enable this type of anticipation and recognition of tentative clues, where the search images, probing, conjectures and hypotheses of organisms—independent of the psychophysical characteristics of the cues (as measured by, say, their vicinity, proximity or size)—can shape judgment and decision making. Thus, again, our approach is firmly focused on the active, *presentational* aspects of rationality, rather than their representational nature.

In a generative sense, awareness toward a cue or cues needs to be actively nurtured—the *relevant* cues need to somehow be identified, presented and made salient, from amongst the meaningless mass of potential and indefinite things within an environment or scene. Returning briefly back to our short Sherlock dialogue, notice how even *after* Sherlock points the dog out to the Scotland Yard detective, the latter still remains puzzled as to why the dog is in any way relevant to the situation, that is, why the dog (cue) represents a “curious incident.” This indeed is the problem: any cue could be “curious” and important, or not. But for something to “pop out” and become meaningful, from amongst indefinite potential cues—or put differently, for a cue to count as evidence, for it to signal something—requires a top-down mechanism. In essence, we are saying that there are indefinite varieties of signal detection beyond simply looking at the amount or intensity of a cue or cues. Our generative form of visual “pop out” therefore is fundamentally different from psychophysical approaches to vision and perception (see Wolfe and Horowitz, 2017). Some form of top-down rationale is needed to enable us to recognize a cue in the first place, as the cue does not inherently impose itself onto our awareness, but only becomes salient in response to active probing. Top-down factors or reasons play a critical role in presenting, specifying and selecting the relevant cues—again, independent of the physical qualities of cues. And in the case of our Sherlock example, the top-down imposition of a “plot”—an imagined, hypothesized conjecture or narrative of what happened—directs salience toward certain objects, cues, features and aspects of the environment (Koenderink, 2012). The plot makes the cue salient. Without a top-down plot, there is no reason whatsoever for the non-barking dog to be salient or evident in any way. It’s only with the top-down plot that a cue (or clue), such as the dog not barking, can even meaningfully be identified.

To offer a contrast, in our hypothetical Sherlock situation it’s hard to point to any of the heuristics from ecological rationality that might similarly resolve the situation. We might, perhaps—*in retrospect*—be able to shoe-horn an explanation that is in line with ecological rationality by saying that the “non-barking-dog”-cue is identified through some mechanism of random or other form of sampling (Though it’s hard to imagine how one might, in the first place, become aware of the non-barking dog and its importance). Or we might highlight a growing “tally” of cues that increasingly, in the aggregate, point to a threshold conclusion that a particular individual is the sought-after culprit in the case—the non-barking-dog being one of many cues pointing in this direction. But any heuristics or associated statistics that we might point to are merely an after-the-fact epiphenomenon of a process that is necessarily initiated top-down. Again, cues themselves don’t say or mean anything, they aren’t somehow inherently evident (based on, say, their physical characteristics). Rather, cues become cues-*for*-something, or clues, in the context of a particular top-down plot. That said, we of course recognize that the plot might be wrong, but it can readily be amended if the relevant cues and evidence cannot be found. Thus we need an *a priori* way of generating awareness toward specific cues, a reason for growing or elevating—and creating salience for—a particular cue based on some top-down factor.

Now, we have of course pointed to a fictional example. But this idea of having a top-down “plot” might be generalized to both mundane, daily experiences as well as more novel ones. To offer an everyday example (linked to the aforementioned example of lost keys): if I have lost my house keys, my visual search for them is guided by a key search image. I know what I am looking for, what my keys look like, and thus I can scan for key-like items in my surroundings. Importantly, this visual “investigation” and search is critically enabled by me having a conjecture or hypothesis (an informal plot, of sorts) about where I might have lost the keys in the first place. I might remember having had the keys two hours earlier, and I might therefore trace my steps and search across the rooms I’ve occupied during the intervening time period. No form of random sampling or item-by-item inspection makes sense in this situation. Nor does any notion of psychophysical salience. After all, not only are my keys “small” but they might have slipped into the crack of the couch and thus not even be visible. But a top-down plot or hypothesis enables me to find them.

Beyond the mundane search for keys, these top-down factors are also the underlying mechanism behind the emergence of novelty, including in the sciences. Science itself might be seen as an effort to “grow” awareness and salience toward novel objects or unique observations, things that previously were non-obvious and seemingly hidden. Theories serve a top-down plot-like function in enabling us to observe and see a new cue, data point or piece of evidence—or to see something (like an apple falling) in a completely new way. As put by Einstein, “whether you can observe a thing or not depends on the theory which you use. It is the theory which decides what can be observed” (Polanyi, 1971, p. 604). Furthermore, theories might lead us to construct instruments or technologies—such as telescopes or microscopes—and methods for making observations of things that are not evident to the naked eye (for a recent discussion, see Felin et al., 2021). For example, the postulation of gravitational waves led to the construction of detectors to measure them. Cue-first-based, psychophysical approaches do not offer this type of mechanism for observing novel things. Bayesian approaches similarly are unable to address questions of novel observation. This is informally illustrated by the fact that no amount of watching falling items (like apples) will yield insights about gravity, without first having a conjecture, hunch or theory about what one is looking for and at.

The idea of top-down theories also has critical implications for economic settings, which abound in uncertainty and latent possibility. Biological intuition has traditionally been applied to economic settings at the level of randomness and environmental selection (Penrose, 1952). Or ecological rationality focuses on the long-run evolutionary adaptation of the mind to changing environments (Gigerenzer, 2000; also see Cosmides and Tooby, 2013). What is missing in this work is the organism-directed probing and exploration that also shapes and creates novelty. That is, rather than merely passively adapting to their surroundings, organisms (including economic agents) make novel use of objects around them. Economic environments are inherently “unprestatable,” and entrepreneurs and managers can identify novel uses and affordances (Kauffman, 2014). Thus, rather than merely adapting to environments, important exaptive mechanisms also play a role (e.g., La Porta et al., 2020; Cattani and Mastrogiorgio, 2021).

## Some Concluding Remarks: Scissors Revisited

We believe that our generative view of rationality offers a unique way to think about rationality, with novel implications for future work. To illustrate this, by way of some concluding remarks, and to highlight links to bounded rationality, we briefly revisit Simon’s (1990) famous and oft-quoted “scissors” metaphor (e.g., Chase et al., 1998; Gigerenzer and Selten, 2001; Gigerenzer and Gaissmaier, 2011; Puranam et al., 2015; Petracca, 2021). Simon’s scissors metaphor is an evocative idea that has been discussed or mentioned in hundreds of articles over the past decades. We highlight how a focus on generativity might offer a useful and different way to think about the two “blades” of the scissors, with attendant implications for judgment and decision making in situations of uncertainty.

Simon’s (1990, pp. 7–9) scissors metaphor is the idea that rationality is shaped by two blades, namely, the “structure of the environment” and the “computational capabilities of the actor.” In the ecological rationality literature, the two blades are summarized as the “internal and external *constraints*” of judgment and decision making (Kozyreva and Hertwig, 2021, p. 1524, emphasis added). Or to cite Todd and Gigerenzer (2003, p. 143), the scissors metaphor is the overarching idea “that human rationality is bounded by both internal (mental) and external (environmental) constraints” (also see Gigerenzer and Goldstein, 1996; Chater and Oaksford, 1999). This two-pronged, blades approach is also central for ongoing definitions of uncertainty as well. For example, Kozyreva and Hertwig (2021, p. 1525) argue that “uncertainty concerns environmental constraints as well as computational constraints, which both prevent the subject from determining the structure of the environment.” In all, the emphasis on both organism-related and environmental *constraints* is ubiquitous and offers a useful contrast to how generative rationality characterizes the two blades.

Rather than focus on constraints (important as they undoubtedly are), our emphasis in this paper has been on the *generative* nature of organisms and the *teeming* nature of environments. Thus our arguments might be seen as a friendly amendment for how we might think about the organism-environment interface—specifically, a call to recognize the novel and emergent aspects of both sides of the organism-environment interface. While the ideals of optimization and constraint are heavily emphasized and juxtaposed in existing work, this has come at the expense of understanding how novelty emerges. Of course, in shifting the emphasis from constraint and boundedness to generativity, we certainly do not mean to suggest—as the examples below will illustrate—that organisms are characterized by some form of omniscience, or that there aren’t costs and limits associated with judgment and decision making. Constraints and boundedness are important. However, we do think that the heavy emphasis on the constraints of information processing—and the experiments constructed to point this out—have unnecessarily sidelined the generative nature of organisms and the possibilities presented by teeming environments.^16^

Before offering some examples, it’s important to point out that the scissors metaphor was specifically discussed by Simon (1990; cf. Newell and Simon, 1972) in an article that focuses on the “invariants” and similarities between human judgment, computers and general information processing (also see Simon, 1980). The computational logic has readily lent itself to extensions like the idea of humans as “intuitive statisticians” and the importance of the statistical toolkit and environmental structure. But this conception of rationality is highly dependent on the types of tasks, experiments and examples that scholars construct and focus on. Computers undoubtedly perform computational tasks well, indeed, better than humans. But what the computational and statistical analogies miss is the situations, tasks and settings where human judgment readily outperforms any form of computation or statistical processing (cf. Culberson, 1998). This is particularly the case for novel situations and uncertain environments, where environmental structure can’t be specified ex ante. For example compared to computers, humans and other living organisms routinely solve the “frame problem,” an impossibility for computers (McCarthy and Hayes, 1981), where humans readily discover new uses and affordances that simply aren’t computationally pre-statable (Felin et al., 2014; Kauffman, 2014).

To make this point more concrete, and to informally contrast the computational logic with the generative one, consider a simple search problem like the frequently discussed search for a needle in a haystack (see Simon, 1969; Simon, 1978; cf. Baumol, 1979; Winter, 2000). Here we have a quintessential (albeit stylized) search problem, where we are faced with an overwhelming search task. To find the needle, we might engage in some form of “brute force” search, where we select an item randomly, and iterate item-by-item through the objects until we encounter the needle or the item we seek (Culberson, 1998). This type of “exhaustive” search of course is overly costly and prohibitive. Thus we might think about applying heuristics or “search rules” to solve the problem—rules about where to search and when to stop searching (see Gigerenzer and Gaissmaier, 2011, pp. 454–456). Simon for example imagines a haystack where needles of varied sharpness are distributed randomly, and highlights how we might decide to satisfice and end search when we encounter a needle that is “sharp enough” (Simon and Kadane, 1975).

But humans can readily solve these types of search problems—like the needle-haystack problem—in various novel and creative ways. For example, we might postulate that the needle is made of steel and is nickel-plated, and therefore use a powerful magnet to quickly find the needle. Or we might, say, burn the haystack or use some kind of large sieve or leaf blower. Or perhaps some kind of sorting device could even be constructed from the hay itself. Or we might delimit the search by hypothesizing that the needle—due to its relative size and weight—is best found by looking on the ground (Felin and Kauffman, 2021). Thus the brute or exhaustive search option need not be held up as an ideal, as varied shortcuts and solutions can be generated. Notice that this type of creative problem-solving—the hallmark of generativity—is in fact ubiquitous in nature. This type of creative problem solving is not just a human prerogative, but innovative problem solving and tool use is evident across species (Fragaszy and Liu, 2012; Griffin and Guez, 2014; Morand-Ferron et al., 2016; Fragaszy and Mangalam, 2018; Amici et al., 2019).

Thus the hacks and solutions to search, judgment and decision making might involve utilizing tools and objects in our environments in various creative ways, beyond statistical inference or computation. Even ecological rationality’s popular city size task (Gigerenzer and Goldstein, 1996)—discussed by us extensively above—can easily be solved by, say, asking someone, or by quickly looking the answer up on the internet. In other words, *in the real world* we use the material resources, affordances and technologies around us in creative ways to come up with solutions (Uexküll, 2010; Gabora, 2019). While the prototypical decision tasks and environments of ecological rationality try to offer a tractable microcosm for helping us understand judgment and decision making, it’s hard to see how these decision tasks—like the frequently used city size experiment—generalize to more uncertain settings. For example, the tasks of an entrepreneur or manager are fundamentally different from anything like comparing city sizes: they are highly ambiguous and highly multidimensional. This doesn’t mean that judgment should be studied by, say, using inkblots. But the classic literature, for example, on functional fixedness (James, 1890; Duncker, 1945), might offer a basis for exploring judgment decision making and creativity in situations of uncertainty.

In all, the existing literature—within the domain of bounded and ecological rationality—should recognize the affordances, uses and functions of the material world. With our focus on the “generative” nature of rationality we hope to emphasize the possibility of these emergent and novel outcomes. The statistical and computational tasks that characterize the extant literature on bounded and ecological rationality are of course important. Undoubtedly representational and statistical approaches have their place. But it’s important for scholars to also address the generative (presentational or even “expressive”) aspects of perception, as these relate to judgment and decision making. Thus our hope is that this paper—an effort to outline the broad contours of a generative approach to rationality—might offer the basis for future work along these lines.

## Data Availability Statement

The original contributions presented in the study are included in the article/supplementary material, further inquiries can be directed to the corresponding author/s.

## Author Contributions

TF wrote the first version of the manuscript, with key ideas and inputs provided by JK. Both authors edited, added to and revised the full manuscript.

## Conflict of Interest

The authors declare that the research was conducted in the absence of any commercial or financial relationships that could be construed as a potential conflict of interest.

## Publisher’s Note

All claims expressed in this article are solely those of the authors and do not necessarily represent those of their affiliated organizations, or those of the publisher, the editors and the reviewers. Any product that may be evaluated in this article, or claim that may be made by its manufacturer, is not guaranteed or endorsed by the publisher.

## Footnotes

1 We certainly recognize that each of these three descriptors—small, non-obvious, and hidden—suggest ontologically different properties. Size (for example, something being comparatively “small”), might be said to essentially deal with physics and physical properties, “hidden” points to perception and Gestalt, and “non-obvious” deals with apperceptive processes. We simply highlight that the generalized emphasis on salience due to psychophysical factors misses critical cues that seemingly have none of these qualities (cf. Koenderink, 2012).

2 The focus on cues and environmental structure are ubiquitous in the ecological rationality literature. For cue-based definitions of environments, see Todd and Gigerenzer (2012), Hertwig et al. (2021), and Kozyreva and Hertwig (2021).

3 The focus on cues and their physical characteristics is equally important in other literatures within psychology. For example, person-situation research focuses heavily on cues by arguing that “the situation consists of objectively quantifiable stimuli called cues” (Rauthmann and Sherman, 2020, p. 473). Cues in this literature are similarly defined as the “physical or objective elements that comprise the environment,” and again, the literature further argues that “they [the cues] can be objectively measured and quantified” Rauthmann et al. (2014, p. 679; cf. Todd and Gigerenzer, 2020).

4 Arguably, the most common reaction of a biological organism to structures where there is “lots” of a cue is probably to ignore these cues, since they can be taken for granted. In this sense, the most useful cues are necessarily rare.

5 Our goal by no means is to dismiss Fechner’s important and voluminous work. We merely point out how the underlying logic of Fechner’s *Elements of Psychophysics*—where cues are taken as givens (and salience is a function of statistical or physical qualities)—has been problematically applied in the context of ecological rationality.

6 We have focused on the early foundations of psychophysics, though arguably a broader conception of “modern” psychophysics would include many important contributions and additions (Kingdom and Prins, 2016; also see Lu and Dosher, 2013). Our goal is not to review this very large literature. Rather, we simply seek to point out how some of the key aspects of psychophysics (the emphasis on the statistical and physical nature of cues) have been misapplied in the context of ecological rationality.

7 For example, standing at Times Square we might observe cues (or signals) like people walking from a certain direction with shopping bags and thus make inferences about a certain shop in that direction. Or to offer another example, the presence of a “yellow” car might signal a taxi. Visual scenes thus abound with varied signals, affordances and meanings that cannot be accounted for through a strict psychophysical lens that is focused on a purely statistical or physical reading of the environment.

8 We should recognize that while ecological rationality focuses on “statistical properties of the environment that exist *independent* of a person’s knowledge” (Kozyreva and Hertwig, 2021, p. 1519, emphasis added), existing work has *rhetorically* (though not substantively) recognized organism-dependence. To illustrate, in his foundational 1956 paper “rational choice and the structure of the environment,” Herbert Simon mentions that “we are *not* interested in describing some physically objective world in its totality, but only those aspects of the totality that have relevance as the ‘life space’ of the organism considered” (Simon, 1956, p. 130, emphasis added). However, the underlying models of search and bounded rationality are organism-independent and general (see Simon, 1980, 1990; for a review, see Felin et al., 2017; also see Chater et al., 2018).

9 Our organism-centric, biologically informed approach here argues that some measure of generativity is needed to account for the ongoing novelty and heterogeneity we observe all around us, whether in nature or in economic settings. Organism-environment interactions are not just a one-way street, where organisms adapt to their environments over time. Organisms also actively shape their environments. Organisms “are agentive and thus capable of initiating activity by themselves” (Longo et al., 2015, p. 5; cf. Noble, 2015).

10 As noted by Albertazzi et al. (2010, p. 8), “the central idea in Brentano’s work, that of perception as presentation, has been entirely missing from cognitive science and has only recently been introduced into contemporary dialogue.” For further discussion of the critically important, phenomenological aspects of vision (including associated neural mechanisms), see Koenderink (2012).

11 The language of “top-down” is occasionally used in the context of the bounded and ecological rationality literatures (e.g., see Todd and Brighton, 2016). However, these appeals to top-down mechanisms still emphasize *predefined* cues, while our specific emphasis is on emergent cues and their psychogenesis (Koenderink, 2012).

12 One exception to this might be the notion of “criterion” that is often mentioned in the context of ecological rationality. However, ecological rationality focuses on how “available cues predict the criterion” (Kozyreva and Hertwig, 2021, p. 1530; also see Hogarth and Karelaia, 2007), while our emphasis instead would be on how a criterion (like a specific question or hypothesis) enables the presentation of relevant cues—a critical distinction.

13 The language of “growing awareness” has also been used in the economics literature (see Karni and Vierø, 2013). However, that literature builds on various large and small-world conceptions (cf. Savage, 1950) to model “expanding state spaces” and their implications for economic decision makers. Our approach, instead, is focused on perception. We address how awareness toward novel cues or objects might be endogenously grown, as well as the critical cue-to-clue transformation (building on Koenderink, 2012).

14 The exploratory and generative process of hypothesizing can be seen as a general biological process, where organisms (of all stripes) engage in this process (Riedl, 1984; cf. Popper, 2013).

15 We use the language of a “cue-to-clue transformation” to make our point about how awareness toward something/anything requires active probing on the part of the organism. In an important sense, the specification or recognition of *any* cue necessarily requires some mechanism for generating awareness. That is, strictly speaking, any qualifier that we might use in front of the word cue (a *salient* cue, a *relevant* cue, an *important* cue, a *meaningful* cue, a *surprising* cue, etc.) is redundant (Koenderink, 2012). However, we nonetheless use this language to help us explicate our central argument relative to existing ecological approaches.

16 There are some research streams that touch on related issues (though they are not directly focused on perception and rationality). For example, Grandori (2010) discusses how the bounded rationality literature also needs to understand scientific and economic *discovery*. Others have focused on notions such as “creative rationality,” and the logic of abduction (e.g., Gooding, 1996; Forest, 2017). Felin and Zenger (2017) look at how economic *theories*—and associated problem formulation and solving—shape perception and the emergence of novelty. More broadly, Viale (2020) recently highlights various literatures that touch on the creative or novel aspects of bounded rationality. Unfortunately we cannot cover all of this work. While all of this work is broadly related, our specific focus is different. Namely, we are focused on the *perceptual* foundations of ecological rationality (as well as our generative alternative). But we certainly see opportunities for future work to carefully make linkages across our arguments and the aforementioned literatures.
